# C‐Cell Carcinoma in a Common Marmoset (*Callithrix jacchus*) With a Brief Review of Thyroid Neoplasms in Neotropical Primates

**DOI:** 10.1111/jmp.70051

**Published:** 2025-11-30

**Authors:** Isabel Luana de Macêdo, Nicole Lynn Gottdenker, Lilian J. Oliveira, Davi Emanuel Ribeiro de Sousa, Yasmin Nunes Godoy da Fonseca, Liz de Albuquerque Cerqueira, Betânia Pereira Borges, Márcio Botelho de Castro

**Affiliations:** ^1^ Graduate Program in Animal Science University of Brasília Brasília Federal District Brazil; ^2^ Veterinary Pathology and Forensic Laboratory, Campus Darcy Ribeiro University of Brasília Brasília Federal District Brazil; ^3^ Department of Pathology, College of Veterinary Medicine University of Georgia Athens Georgia USA; ^4^ Brasília Zoo Foundation Brasília Brazil

## Abstract

A managed‐care female common marmoset developed a rare C‐cell carcinoma in the right thyroid lobe, characterized by capsular and intrathyroidal invasion. Neoplastic plasmacytoid cells arranged in sheets exhibited a morphological and immunophenotypic profile (calcitonin+, chromogranin A+, CK7+, INSM1+, TTF‐1) that closely resembles human thyroid C‐cell tumors.

## 1 Introduction

Endocrine proliferative lesions, including hyperplasia and neoplasms, present a diagnostic challenge across all species [1, 2, 3]. Histomorphology alone often is not specific and requires hormonal profiling and a targeted panel of immunohistochemical markers [4]. Endocrine tumors have been reported primarily in macaques and are uncommon in nonhuman primates (NHPs) of the Platyrrhini order, mostly reported in the adrenal glands [5]. Thyroid neoplasms, although rare in NHPs [6], have also been found predominantly in Catarrhini, with a few cases arising from follicular cells [5].

Thyroid C‐cell tumors are sporadic in NHPs, with one case reported in a macaque [7]. In humans, tumors of parafollicular cells are primarily classified as medullary thyroid carcinoma (MTC) or C‐cell adenoma [8]. Due to the histological classification complexity of endocrine tumors, immunohistochemistry (IHC) is an essential tool for confirming cellular origin and distinguishing between hyperplastic and neoplastic processes, such as in thyroid proliferative lesions [9].

This report describes the pathological and immunohistochemical features of a medullary thyroid carcinoma (C‐cell carcinoma) in a managed‐care common marmoset ( *C. jacchus* ), aiming to contribute to the knowledge of this very uncommon endocrine neoplasm in NHPs.

## 2 Case Report

**FIGURE 1:**
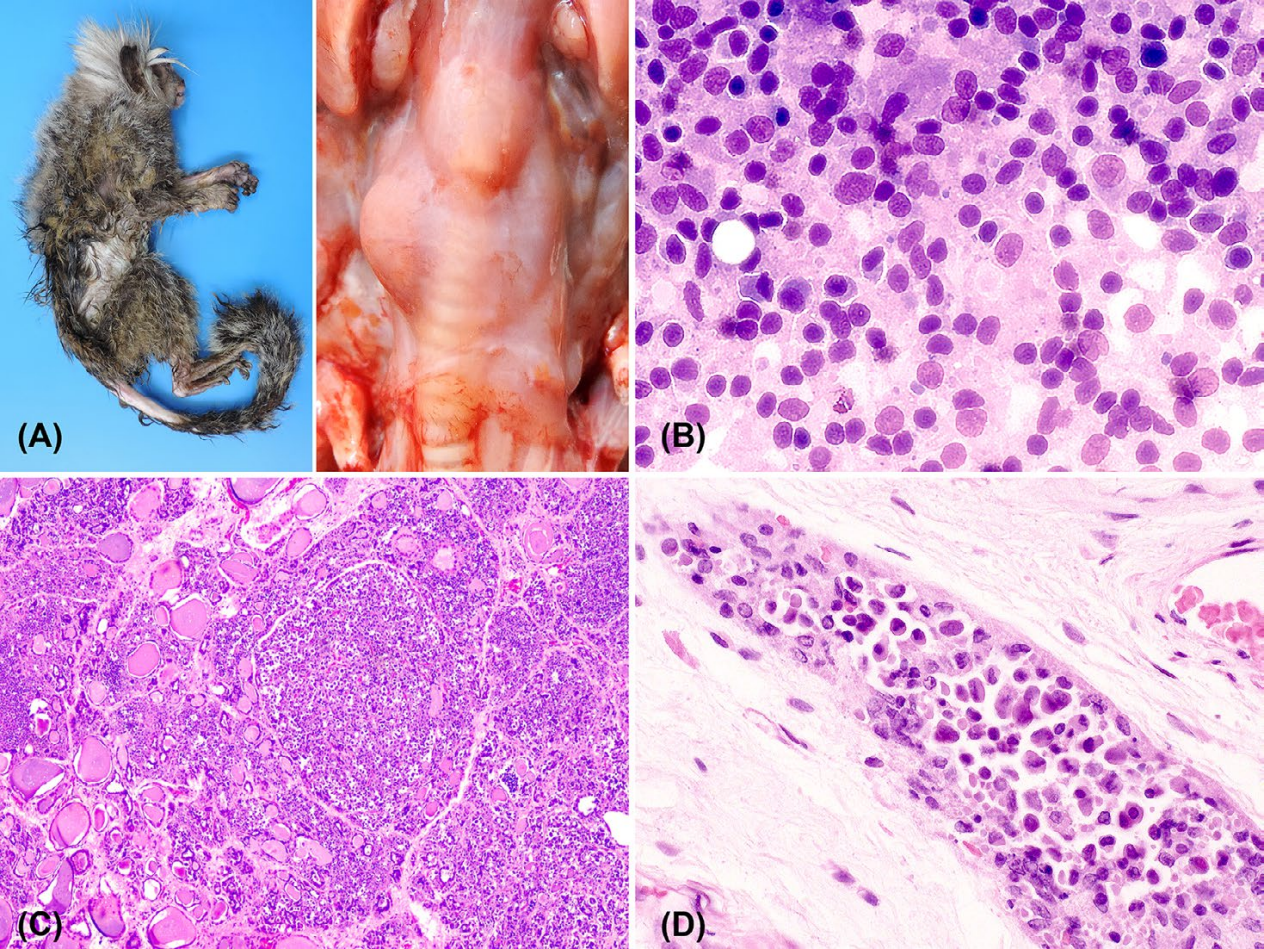
C‐cell carcinoma in a common marmoset ( *Callithrix jacchus* ). (A) Left: Marmoset with poor body condition. Right: Asymmetrical enlargement of the thyroid glands; the right thyroid gland is markedly larger than the left. (B) Cytological features of medullary thyroid carcinoma (MTC): clusters of round cells exhibiting moderate pleomorphism. Papanicolaou stain, objective ×40. (C) Thyroid gland parenchyma expanded and effaced by a nonencapsulated infiltrative neoplasm. Hematoxylin and eosin (H&E), objective ×10. (D) Capsule infiltrated by cells with plasmacytoid/epithelioid. H&E, objective ×40.

An adult female common marmoset ( *C. jacchus* ) from the Brasília Zoo Foundation collection was found dead in the facility and taken for an autopsy at the Veterinary Pathology and Forensic Laboratory of the University of Brasília. The marmoset was severely cachectic, weighing 132.2 g (Figure 1A, left). Gross examination showed marked hepatobiliary parasitism by *Platynosomum illiciens*. Additionally, the right thyroid gland was enlarged with a well‐demarcated, bulged, pale, and firm nodule measuring 1.0 × 0.5 × 0.5 cm (Figure 1A, right). A fine needle aspiration cytology (FNA) during the autopsy demonstrated high cellularity, cellular pleomorphism, discohesive neoplastic cells with a plasmacytoid or epithelioid pattern, a high nuclear/cytoplasmic ratio, some binucleation or multinucleation, coarse chromatin, and one or more nucleoli (Figure 1B).

**FIGURE 2:**
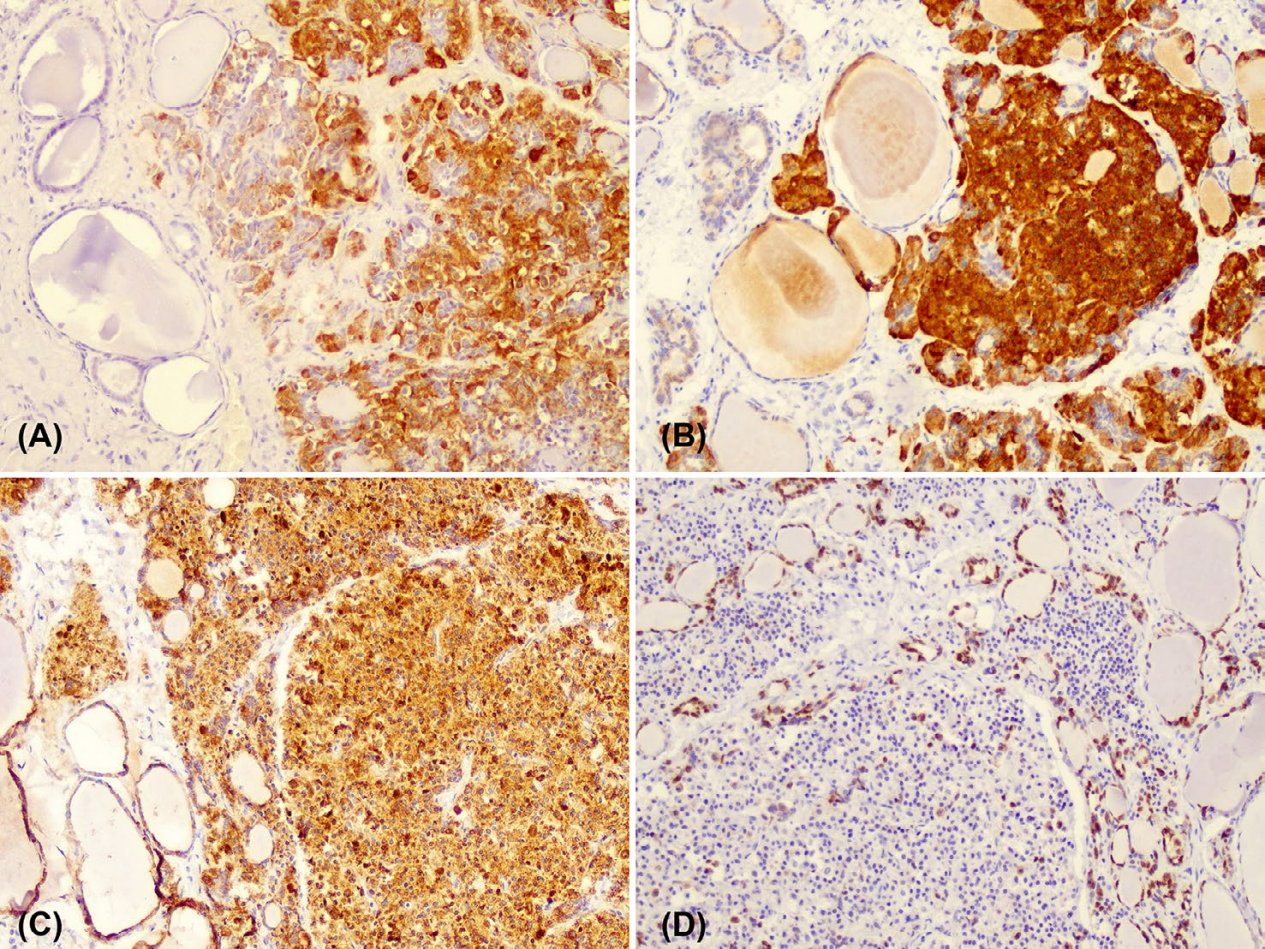
Immunohistochemical features of C‐cell carcinoma in a common marmoset ( *Callithrix jacchus* ). Positive immunolabeling for calcitonin (A), chromogranin A (B), and CK7 (C), and negative immunolabeling for TTF‐1 (D). Immunoperoxidase, objective 20×.

Tissue samples collected in the autopsy were fixed in 10% neutral‐buffered formalin, routinely processed, embedded in paraffin, sectioned at 5 μm, and stained with hematoxylin and eosin (H&E) for histological evaluation. Histologically, the right thyroid gland parenchyma was expanded and effaced by a nonencapsulated infiltrative neoplasm (Figure 1C) composed of plasmacytoid/epithelioid cells, with some multinucleated cells arranged in dense sheets and nests that compressed the adjacent parenchyma and also infiltrated the thyroid fibrous capsule. Neoplastic cells had moderate anisocytosis, anisokaryosis, round to oval and centrally oriented nuclei with coarse chromatin, one or more prominent nucleoli, and variable amounts of eosinophilic cytoplasm with variable cell borders (Figure 1D). There were two mitotic figures in fifteen FN18/40× fields (2.37 mm^2^), and small, scattered areas of necrosis were present within the neoplasm parenchyma. IHC was performed using 4plus Streptavidin HRP Label from Biocare Medical and a panel of antibodies (Table S1). The neoplastic cells exhibited strong immunolabeling for calcitonin (Figure 2A), chromogranin A (Figure 2B), and cytokeratin‐7 (CK7) (Figure 2C), as well as moderate nuclear immunostaining for insulinoma‐associated protein 1 (INSM‐1). Additionally, neoplastic cells did not stain for thyroid transcription factor 1 (TTF‐1) (Figure 2D), which supports the diagnosis of MTC. A comprehensive search of Google, PubMed, CABI Direct, Web of Science, and Scopus, using search terms “non‐human primates”, “endocrine tumors”, “thyroid tumors”, “neotropical primates”, “new world monkey”, “*Callithrix*”, “*Sapajus*”, “*Aotus*”, “*Ateles*”, “*Alouatta*”, “*Saguinus*” did not yield any reports of MTC in common marmosets. The few case reports on follicular thyroid tumors are described in Table S2.

## 3 Discussion

This brief review demonstrated thyroid gland neoplasms are uncommon, with no reports of C‐cell neoplasms in neotropical NHPs, and with only one case of MTC in a managed‐care Rhesus macaque [7]. In humans, MTC accounts for only 5%–10% of all thyroid malignancies [10]. To the best of our knowledge, this case represents the first report of C‐cell thyroid carcinoma in a common marmoset ( *Callithrix jacchus* ).

In this study, the marmoset showed no clinical signs, apart from cachexia, which was attributed to the severe hepatic platynosomosis [11] rather than to the C‐cell carcinoma. Similarly, no clinical signs were reported in a case of MTC in a macaque [7]. In general, NHPs with thyroid neoplasms are older and asymptomatic regarding tumor‐related manifestations [12, 13, 14, 15], suggesting this case of MTC in the marmoset represented an incidental finding.

In this case, the cytopathological features of C‐cell carcinoma, characterized by discohesive neoplastic cells and occasional multinucleated cells, were very similar to those described in humans [16] and may serve as an ancillary aid for diagnosis in NHPs practice. Histologically, infiltrative neoplastic cells with distinct plasmacytoid to epithelioid morphology, as observed in the marmoset, exhibit a similar growth pattern in human cases of MTC [8, 17], and less frequently in animals [18, 19]. Neoplastic capsular and intrathyroidal invasion and scattered foci of necrosis were similar to the histologic criteria of malignancy for MTC in other species and humans [8, 17, 18, 19], confirming the malignant nature of the thyroid neoplasm in the marmoset.

The immunopositivity of neoplastic cells for calcitonin, chromogranin A, CK7, and INSM1, combined with a negative result for TTF‐1, supported the final diagnosis of MTC in this case. In humans, neoplasms from C cells typically express calcitonin and neuroendocrine markers such as chromogranin A and INSM1, alongside variable expression of CK7 [20, 21]. TTF‐1, while often expressed in follicular‐derived thyroid carcinomas and pulmonary neuroendocrine tumors, is usually absent in MTC [22]. These findings suggest that similar immunohistochemical markers can be reliably used to identify and characterize thyroid C‐cell neoplasms in NHPs.

The differential diagnoses for MTC include primary thyroid lesions such as C‐cell hyperplasia and C‐cell adenoma [9, 23], which were excluded in this case due to the clear malignant features observed, including capsular invasion. Follicular adenoma or adenocarcinoma [9, 24, 25, 26] were also ruled out based on negative TTF‐1 immunostaining. Additionally, metastatic tumors should be considered as differentials, such as lymphoma or other round cell neoplasms. Nevertheless, the expression of calcitonin and chromogranin A ruled out the possibility of metastatic disease in this marmoset.

These findings highlight, despite their rarity, the spontaneous occurrence of thyroid C‐cell carcinomas in a marmoset and their potential contribution to comparative medicine. MTC in neotropical primates likely shares characteristics with cases described in humans and other veterinary species, providing valuable insights that may enhance diagnostic accuracy and understanding of its biological behavior.

## Funding

This work was supported by Coordenação de Aperfeiçoamento de Pessoal de Nível Superior.

## Ethics Statement

This study did not involve the use of live animals. The necropsy was performed on a zoo‐housed nonhuman primate that was found dead and submitted for diagnostic investigation as part of the Brazilian Yellow Fever surveillance program. All procedures complied with institutional ethical standards and national regulations for the use of animal biological material. Ethical approval was not required, as no experimental procedures or interventions were performed.

## Conflicts of Interest

The authors declare no conflicts of interest.

## Supporting information


**Table S1:** Antigens, immunolabeling for primary antibodies, and dilutions applied to the thyroid neoplasm.

**Table S2:** Review of thyroid tumors in neotropical NHPs, summarizing species, age, sex, type of neoplasm, signalment, gross lesions, histopathological features, immunohistochemical markers, and references.

## Data Availability

Data sharing not applicable to this article as no datasets were generated or analyzed during the current study.
