# Supplementary material for: C‐Cell Carcinoma in a Common Marmoset (Callithrix jacchus) With a Brief Review of Thyroid Neoplasms in Neotropical Primates
Source: J Med Primatol. 2025 Nov 30;54(6):e70051. doi: 10.1111/jmp.70051 (PMC12665875; doi:10.1111/jmp.70051)
Supplement: Supplementary file 1 — Table S1: Antigens, immunolabeling for primary antibodies, and dilutions applied to the thyroid neoplasm. [file JMP-54-e70051-s002.docx]

**Table S1.** Antigens, immunolabeling for primary antibodies, and dilutions applied to the thyroid neoplasm.

| Primary Antibody | Manufacturer | Clone number | Positive control^º^ | Antigen retrieval^*,◊,§,♦^ | Dilution/incubation | Immunostaining^★^ |
| --- | --- | --- | --- | --- | --- | --- |
| Calcitonin | Dako Corp. | A0576 | Thyroid | Citrate – 15 min | 1:2000/60 min | +^a^ |
| Chromogranin A | Bio SB | LK2H10 | Pancreas | Citrate – 15 min | 1:100/60 min | +^a^ |
| CK7 | Abcam | OV-TL12/30 | Skin | High pH 9.0 – 15 min | 1:400/30 min | + ^a^ |
| INSM-1 | Santa Cruz Biotech. | A‑8 | Neuroendocrine tumor | High pH 9.0 – 15 min | 1:100/60 min | +^b^ |
| TTF-1 | Dako Corp. | 1A5 | Lung | Citrate – 15 min | 1:500/60 min | - |

CK7: Cytokeratin 7; INSM-1: Insulinoma-associated protein 1; TTF-1: Thyroid transcription factor 1; ^º^canine tissues; ^*^ 110ºC, pressure cooker; ^◊^Peroxidase block: 3% H_2_O_2_, 5 min; ^§^Protein block: Power block (Biogenex), 5 min; ^♦^Chromogen: 3,3′-diaminobenzidine (DAB), 12 min. ^★^Immunostaining: + positive; - negative; ^a^ cytoplasmic immunolabeling; ^b^ nuclear immunolabeling.
