# Supplementary material for: C‐Cell Carcinoma in a Common Marmoset (Callithrix jacchus) With a Brief Review of Thyroid Neoplasms in Neotropical Primates
Source: J Med Primatol. 2025 Nov 30;54(6):e70051. doi: 10.1111/jmp.70051 (PMC12665875; doi:10.1111/jmp.70051)
Supplement: Supplementary file 2 — Table S2: Review of thyroid tumors in neotropical NHPs, summarizing species, age, sex, type of neoplasm, signalment, gross lesions, histopathological features, immunohistochemical markers, and references. [file JMP-54-e70051-s001.docx]

**Table S2.** Review of thyroid tumors in neotropical NHPs, summarizing species, age, sex, type of neoplasm, signalment, gross lesions, histopathological features, immunohistochemical markers, and references.

| **Species, age, sex** | **Neoplasm** | **Clinical signalment** | **Gross findings** | **Histopathology Features** | **IHC Markers** | **Reference** |
| --- | --- | --- | --- | --- | --- | --- |
| *Callithrix jacchus*  10yo male | Follicular adenoma | No clinical abnormalities reported. | Left thyroid lobe was enlarged (2.0x1.5x1.0 cm). | Encapsulated mass compressing the normal thyroid tissue. Composed of variably sized and irregularly shaped thyroid follicles lined by a single layer of columnar epithelial cells with tubular or papillary proliferation. And accumulation of the basement membrane. | Collagen IV (+) in BM. | (Kawasako et al., 2014) – (13) |
| *Callithrix jacchus*  Not reported | Follicular adenoma | Not reported. | Not reported | Incidental benign tumor  (not described) | – | (Dias et al., 1996) – (12) |
| *Mico melanurus*  4y11mo female | Papillary thyroid cystadenoma | Abnormal behavior and canine tooth abscess. Died due to *Pasteurella multocida* septicemia. | No remarkable findings | Well-demarcated and encapsulated papillary proliferation lined by well-differentiated cuboidal cells within a cystic formation filled with proteinaceous fluid. | – | (Dias et al., 1996) – (12) |
| *Mico melanurus*  11y6mo female | Papillary thyroid cystadenoma | Acute onset of diarrhea and died 36 hours later due to a diffuse, severe lymphoplasmacytic gastroenteritis. | No remarkable findings | Multiple cystic formations surrounding papillary proliferations lined by well-differentiated cuboidal to cylindrical epithelial cells. | – | (Dias et al., 1996) – (12) |
| *Saguinus oedipus*  20y7mo female | Papillary thyroid cystadenoma | Gradual weight loss and concomitant pheochromocytoma. | No remarkable findings | Expansile, encapsulated cystic neoplasms that had projections of neoplastic follicular epithelium forming papillary fronds within the cystic cavities. | – | (Miller et al., 2009) – (14) |
| *Saguinus oedipus*  21y9mo male | Papillary thyroid cystadenoma | Gradual weight loss and concomitant pheochromocytoma. | No remarkable findings | Expansile, encapsulated cystic neoplasms that had projections of neoplastic follicular epithelium forming papillary fronds within the cystic cavities. | – | (Miller et al., 2009) – (14) |
| *Saguinus nigricollis*  Adult, female | Follicular carcinoma | Clinically healthy female, pregnant with two. Died following delivery of the second infant. | No remarkable findings | Nests of well-differentiated epithelial cells separated by dense collagenous stroma, with evidence of local invasion into the capsular tissue. | – | (Williamson & Hunt, 1970) – (16) |
| *Saimiri sciureus*  Female | Follicular adenoma | Not reported | Not reported | Typical follicular adenoma, incidental, benign | – | (Lowenstine, 1986) – (26) |
